# Accurate Data Processing Improves the Reliability of Affymetrix Gene Expression Profiles from FFPE Samples

**DOI:** 10.1371/journal.pone.0086511

**Published:** 2014-01-29

**Authors:** Maurizio Callari, Antonio Lembo, Giampaolo Bianchini, Valeria Musella, Vera Cappelletti, Luca Gianni, Maria Grazia Daidone, Paolo Provero

**Affiliations:** 1 Department of Experimental Oncology and Molecular Medicine, Fondazione IRCCS Istituto Nazionale dei Tumori, Milan, Italy; 2 Department of Molecular Biotechnology and Life Sciences, University of Turin, Turin, Italy; 3 Department of Medical Oncology, Ospedale San Raffaele, Milan, Italy; 4 Center for Translational Genomics and Bioinformatics, Ospedale San Raffaele, Milan, Italy; Deutsches Krebsforschungszentrum, Germany

## Abstract

Formalin fixed paraffin-embedded (FFPE) tumor specimens are the conventionally archived material in clinical practice, representing an invaluable tissue source for biomarkers development, validation and routine implementation. For many prospective clinical trials, this material has been collected allowing for a prospective-retrospective study design which represents a successful strategy to define clinical utility for candidate markers. Gene expression data can be obtained even from FFPE specimens with the broadly used Affymetrix HG-U133 Plus 2.0 microarray platform. Nevertheless, important major discrepancies remain in expression data obtained from FFPE compared to fresh-frozen samples, prompting the need for appropriate data processing which could help to obtain more consistent results in downstream analyses. In a publicly available dataset of matched frozen and FFPE expression data, the performances of different normalization methods and specifically designed Chip Description Files (CDFs) were compared. The use of an alternative CDFs together with fRMA normalization significantly improved frozen-FFPE sample correlations, frozen-FFPE probeset correlations and agreement of differential analysis between different tumor subtypes. The relevance of our optimized data processing was assessed and validated using two independent datasets. In this study we demonstrated that an appropriate data processing can significantly improve the reliability of gene expression data derived from FFPE tissues using the standard Affymetrix platform. Tools for the implementation of our data processing algorithm are made publicly available at http://www.biocut.unito.it/cdf-ffpe/.

## Background

Gene expression profiling has proved to be successful in cancer research [1]. The first pivotal studies [2], [3] have been conducted using fresh-frozen samples as source of RNA since commercially available whole gene expression platforms were designed for high quality RNA. The need of fresh-frozen samples has represented one of the major limiting factors in biomarker discovery, development, validation and clinical implementation since few tissue banks with frozen specimens linked to clinical meaningful information were available [4]. On the contrary, formalin-fixed paraffin-embedded (FFPE) tissue specimens are collected for clinical routine diagnostics almost everywhere allowing a broader clinical implementation of biomarkers developed from such source of material. Moreover, archival FFPE tumor blocks retrospectively collected from patients enrolled in prospective clinical trials can be used to generate under specific circumstances the highest level of evidence for the clinical utility of candidate biomarkers avoiding the need to perform expensive and time consuming prospective clinical trials [5]. The commercially available tool Onco*type* DX which is broadly used in the clinical practice for breast cancer patients has been validated so far only using retrospective-prospective studies design [6].

Unfortunately, the age of collected tumor blocks and fixation process induce RNA fragmentation, chemical modifications and RNA-protein cross-linking, making gene expression analysis challenging [7]. Such a limitation has been successfully overcome by using qRT-PCR [6], [8], but this approach limits the number of genes which can be assessed and the potential advantage of testing multiple signatures at a time with one single assay [9]. The availability of technical and analytical methods for generating whole gene expression profile (GEP) from FFPE derived RNA could overcome this limitation, thus providing extremely valuable information. Remarkable examples of this approach are represented by the successful achievement of Affymetrix-based gene expression profiling from tumor samples collected in the NOAH trial [10], [11] and NeoSphere trials [12] and even larger studies are in their planning or execution stages [13].

Specific technologies have been developed to use fragmented RNA, like the Illumina DASL platform [14] and *ad hoc* protocols have been defined to optimize all the analytical steps required to use the Affymetrix GeneChip platforms [15]. Both these platforms proved suitable to yield biologically meaningful GEP data from FFPE clinical samples.

Nevertheless, when gene expression data derived from frozen and FFPE samples are compared, a high level of discrepancy is observed. In a study on diffuse large B-cell lymphoma (DLBCL), Williams et al. [15] processed 59 matched frozen and FFPE samples from patients belonging to two subgroups (activated B-cell, ABC and germinal center B-cell, GCB). In their analysis, 1428 genes were found as differentially expressed (DE) with a false discovery rate (FDR) <5% between the two subgroups using gene expression data generated from frozen samples, whereas only 289 genes were found DE, at the same FDR threshold, when data from FFPE samples were used. Moreover, only 35 of the top 100 DE genes were in common.

Many pre-analytical factors can affect the quality of FFPE-derived expression data, including sample collection and storage procedures and RNA extraction or amplification methods, so that an accurate quality pre-assessment evaluation is needed [16]. Therefore it is natural to expect that the methods applied during the data processing can also affect the downstream results.

Several methods to summarize and normalize expression data were developed for the Affymetrix platforms, with MAS5 and RMA [17] being the most commonly used, and the choice of the normalization algorithm was demonstrated to have a major influence on downstream analysis [18]. A new method called fRMA was recently proposed [19] which, contrary to RMA, allows to normalize microarrays one by one, a useful property in biomarker development studies where the final goal is to classify new patients individually. A peculiarity of the Affymetrix platform is that the expression of each gene is measured by one or more probesets and, for each probeset, the signal is generated by several independent probes. These probes were designed according to sequence annotation available at the time the chip was developed. As the transcript annotation changed over time, probe re-annotation based on updated knowledge of the transcript was shown to improve data quality [20], [21]. This process can be applied by creating alternative Chip Description Files (CDFs) using only the probes matching the most up-to-date gene annotation to assess the expression level.

Expression profiles from FFPE samples are expected to have a lower signal to noise ratio, and an appropriate processing could be crucial to derive a reliable measure of gene expression. At this time, it is not known which processing approach should be used to produce the best results. Indeed, of 15 publications reporting Affymetrix expression data generated from FFPE samples, 4 used MAS5, 6 used RMA and 5 used other or unspecified procedures. None has reported the use of alternative CDFs.

We thus set out to compare the performance of three different summarization and normalization methods, MAS5, RMA and fRMA used in combination with the standard Affymetrix CDF or two different alternative CDFs. The first CDF included all the probes unambiguously mapping RefSeq transcripts, while the second was defined similarly to the first one but contains, for each probeset, only the probes nearest to 3′-end as these probes could be the most informative and reliable in FFPE data because of RNA fragmentation. All these processing pipelines were applied to the Williams dataset [15] evaluating the agreement between matched frozen and FFPE data by several metrics. Findings were further assessed using two independent breast cancer datasets.

## Results

### Generation of alternative CDFs

We developed an alternative CDF, hereafter referred as *RefSeq_all*, for the Affymetrix chips of the HG-U133 series, where all the probes unambiguously mapping RefSeq transcripts were retained and merged to create a new single set of probes for each gene. As result, genes which were represented by multiple probe sets in the original chip design resulted in a new single probeset. A total of 16,991 probesets were generated and about half of them (45.3%) contain more than the 11 probes present in standard Affymetrix probesets (Figure S1A), thus potentially increasing the statistical power in measuring expression levels.

To verify the effect of RNA degradation on probe signals, we focused on the distance from the 3′-end of the 11 probes in each standard Affymetrix probeset in the Williams dataset. Moving toward the 5′, a decay in their signals was observed both in frozen and FFPE data, but the effect was much more striking in FFPE data (Figure S1B). A 3′-bias was indeed expected as a direct consequence of fixation and RNA degradation despite the use of a combination of random primers and oligo-dT in the retrotranscription step. We therefore hypothesized that probes nearer to the 3′-end would be the most informative and reliable, especially in FFPE data.

To verify our hypothesis, after probe re-annotation described before, we computed the distance from the probe to the 3′-end of the transcript. The correlation between frozen and FFPE data turned out to be a decreasing function of such distance, reaching a plateau at around 250–300 bp that corresponds with the average size of aDNA obtained after amplification (Figure S2). Thus we created a second CDF using, for each transcript, only the five probes closest to the 3′-end and in any case mapping within 300 bp from the 3′-end (this alternative CDF was called *RefSeq_dist*). Using these criteria it was possible to define 8,263 probesets, in which the reliability of each probe was likely increased, although the number of probes measuring each gene was reduced.

### Normalization algorithms

Three different normalization algorithms were considered in the subsequent analysis: i) MAS5, ii) RMA [17] and iii) fRMA [19]. In fRMA normalization, the single-sample normalization is achieved by applying pre-computed parameters estimated using a large set of CEL files derived from the Gene Expression Omnibus (GEO) repository [22]. As those parameters need to be re-estimated when using an alternative CDF, we carried out our own estimation using the same pool of publicly available samples used in [19].

### Frozen – FFPE agreement using different processing pipelines

To identify the best performing procedure to process FFPE-derived Affymetrix data, we compared the matched frozen and FFPE GEPs of the Williams dataset [15] after processing the raw data with the three different normalization methods previously described in combination with three different CDFs (the Affymetrix standard CDF, the *RefSeq_all* CDF and the *RefSeq_dist* CDF). From the data processed with the standard CDF, we also extrapolated the subset of probesets mapping on genes targeted in the *RefSeq_all* and *RefSeq_dist* CDFs, in order to undertake a fair comparison. Moreover, we applied the method proposed by Li and colleagues [23] to select the optimal Affymetrix probeset for each gene. We considered the results obtained from frozen samples as the gold standard, and rated the different processing approaches with various measures of concordance between frozen and FFPE samples.

First we evaluated, for each pair of samples, the correlation between frozen- and FFPE-derived expression values over all probesets (hereafter called sample correlation) and, for each probeset, the correlation between frozen- and FFPE-derived expression values over all samples (hereafter called probeset correlation). As not all genes are expected to be expressed or to vary significantly in a specific tissue type, the second analysis was performed only for the 50% most variant probesets (Table 1). Wilcoxon matched pairs test was used to test the significance of the differences in sample correlation between the processing pipelines (Table S1), whereas the differences in probeset correlation were evaluated using the Wilcoxon-Mann-Whitney test (Table S2). Correlations invariably increased when using RMA or fRMA instead of MAS5. The highest median sample correlation was obtained when using the RMA or fRMA normalized data in combination with the *RefSeq_dist* CDF (Figure S2). On the contrary, RMA or fRMA normalized data in combination with the *RefSeq_all* CDF gave the highest probeset correlation. This is in keeping with what observed at probe level, where probes near to 3′-end were those giving the most similar signals in frozen and FFPE data that translates in a higher sample correlation. On the other hand probeset correlation is systematically higher for the Refseq_all CDF. This might be due to the higher number of probes per probeset. Indeed within this CDF frozen/FFPE probeset correlation is significantly associated (r = 0.23, P<2.2e-16) with the number of probes included in the probeset. The simple filtering of the standard probesets using the method by Li et al. [23] gave some improvement compared with the evaluation of all standard probesets but performed worse than *RefSeq_all* CDF.

**Table 1:** Frozen-FFPE correlation analysis for each processing procedure.

CDF	Normalization	Number of probesets	Number of genes	median frozen-FFPE sample correlation	median frozen-FFPE probeset correlation (50% higher IQR)
*CDF standard*	*MAS5*	54675	19798	0.691	0.116
*CDF standard*	*RMA*	54675	19798	0.792	0.333
*CDF standard*	*fRMA*	54675	19798	0.784	0.347
*CDF standard common with RefSeq_all*	*MAS5*	36727	16991	0.709	0.135
*CDF standard common with RefSeq_all*	*RMA*	36727	16991	0.777	0.359
*CDF standard common with RefSeq_all*	*fRMA*	36727	16991	0.773	0.371
*CDF standard common with RefSeq_dist*	*MAS5*	18517	8263	0.713	0.131
*CDF standard common with RefSeq_dist*	*RMA*	18517	8263	0.783	0.360
*CDF standard common with RefSeq_dist*	*fRMA*	18517	8263	0.776	0.371
*CDF standard jetset filtered*	*MAS5*	19178	19178	0.708	0.129
*CDF standard jetset filtered*	*RMA*	19178	19178	0.781	0.370
*CDF standard jetset filtered*	*fRMA*	19178	19178	0.775	0.384
*CDF RefSeq_all*	*MAS5*	16991	16991	0.759	0.228
*CDF RefSeq_all*	*RMA*	16991	16991	0.790	0.420
*CDF RefSeq_all*	*fRMA*	16991	16991	0.782	0.430
*CDF RefSeq_dist*	*MAS5*	8263	8263	0.736	0.178
*CDF RefSeq_dist*	*RMA*	8263	8263	0.795	0.349
*CDF RefSeq_dist*	*fRMA*	8263	8263	0.801	0.359

In the second step we took advantage of the presence of both molecular subgroups of DLBCL in the dataset (ABC and GCB) by performing a class comparison analysis for all processing pipelines, separately for frozen- and FFPE-derived data (Table 2). We computed: a) the correlation (and 95% confidence interval) between fold changes obtained in frozen- and FFPE-derived data; b) the frozen-FFPE fold change slope (and 95% confidence interval); c) the percentage of differentially expressed (DE) probesets observed in frozen samples which were found DE also in FFPE data (i.e. FFPE data sensitivity); d) the percentage of DE probesets observed in FFPE samples which were found DE also in frozen data (i.e. FFPE data positive predictive value). Results, reported in Table 2, showed that, in agreement with the correlation analysis, MAS5 gave the poorest agreement between frozen and FFPE data, independently of the CDF used and for all computed metrics. Consequently, the use of MAS5 algorithm seems to be strongly disadvantageous. fRMA only slightly outperformed RMA but in our opinion it is the preferable option because it implements a normalization method which is applicable independently on each single-sample. This approach allowed for potential clinical applications for which the assessment of tumor samples from patients is made one at a time and also in the context of translational research for which new samples could be added to the ongoing project without introducing potential batch effects associated to the different groups of normalization. The best fold change correlation was obtained for fRMA normalization in combination with the *RefSeq_all* CDF (R = 0.76), corresponding also to the highest percentage of commonly DE probesets (41.7%, about twice the value obtained with MAS5 method and the standard CDF) and increased positive predictive value (69.1%). When using the *RefSeq_dist* CDF, besides the highest sample correlations reported in Table 1, we observed the best frozen-FFPE fold change slope, confirming that the probes nearer to 3′-end are those giving the most concordant signal in frozen and FFPE data. Moreover, looking at the common genes between *RefSeq_all* and *RefSeq_dist* CDFs, the same genes evaluated using only the 5 probes nearest to 3′-end gave on average higher signals (p<2-2e-16, Wilcoxon matched pairs test) in keeping with what observed in the degradation plots (Figure S3). Nevertheless, using the *RefSeq_dist* CDF we did not obtain an improvement in frozen-FFPE probeset correlation (Table 1) nor in the agreement of probesets called as differentially expressed (Table 2). Therefore, in the trade-off between the increasing of the statistical power in measuring the expression levels of each gene (i.e. using the *RefSeq_all* CDF) and the selection of the most reliable probes (i.e. using the *RefSeq_dist* CDF), the first approach seemed to be globally advantageous.

**Table 2:** Frozen-FFPE comparison results for each processing procedure.

CDF	Normalization	Frozen-FFPE Fold change correlation			Frozen-FFPE Fold change slope			Percentage of DE probesets in Frozen data called as DE in FFPE data	Percentage of DE probesets in FFPE data called as DE in Frozen data
		Value	CI lower 5%	CI upper 95%	Value	CI lower 5%	CI upper 95%		
*CDF standard*	*MAS5*	0.407	0.400	0.414	0.442	0.434	0.451	20.7	52.9
*CDF standard*	*RMA*	0.696	0.691	0.700	0.525	0.520	0.529	28.4	59.8
*CDF standard*	*fRMA*	0.717	0.713	0.721	0.627	0.622	0.632	31.5	55.8
*CDF standard common with RefSeq_all*	*MAS5*	0.430	0.422	0.438	0.472	0.462	0.482	23.1	63.2
*CDF standard common with RefSeq_all*	*RMA*	0.706	0.701	0.712	0.543	0.537	0.548	29.8	65.6
*CDF standard common with RefSeq_all*	*fRMA*	0.726	0.721	0.731	0.637	0.631	0.643	32.2	65.4
*CDF standard common with RefSeq_dist*	*MAS5*	0.413	0.401	0.425	0.461	0.446	0.476	20.8	58.7
*CDF standard common with RefSeq_dist*	*RMA*	0.695	0.688	0.703	0.550	0.542	0.558	29.2	62.7
*CDF standard common with RefSeq_dist*	*fRMA*	0.715	0.708	0.722	0.649	0.640	0.659	32.4	64.0
*CDF standard jetset filtered*	*MAS5*	0.423	0.412	0.435	0.450	0.436	0.463	19.4	62.5
*CDF standard jetset filtered*	*RMA*	0.708	0.701	0.715	0.520	0.513	0.527	29.6	69.9
*CDF standard jetset filtered*	*fRMA*	0.732	0.726	0.739	0.626	0.618	0.635	34.0	65.1
*CDF RefSeq_all*	*MAS5*	0.566	0.556	0.576	0.526	0.514	0.537	30.9	63.0
*CDF RefSeq_all*	*RMA*	0.737	0.730	0.744	0.526	0.519	0.533	35.9	67.6
*CDF RefSeq_all*	*fRMA*	0.761	0.755	0.767	0.634	0.626	0.642	41.7	69.1
*CDF RefSeq_dist*	*MAS5*	0.468	0.451	0.484	0.491	0.471	0.511	18.5	62.5
*CDF RefSeq_dist*	*RMA*	0.688	0.676	0.699	0.626	0.611	0.640	26.7	74.2
*CDF RefSeq_dist*	*fRMA*	0.694	0.683	0.705	0.694	0.678	0.709	24.7	70.0

To evaluate the robustness of our results we generated partial datasets by removing one sample at a time, and we evaluated how PPV and sensitivity varied when removing one array (we thank one of the reviewers for suggesting this check). This showed that the effect of array removing is much smaller than the difference between pipelines. For example, when removing in turn each of the 45 classified samples we obtained from MAS5 with standard CDF an average PPV of 0.524 (standard deviation 0.024), while for fRMA with the RefSeq_all CDF we obtained 0.694 (0.025). Similar results apply to the sensitivity.

**Figure 1:**
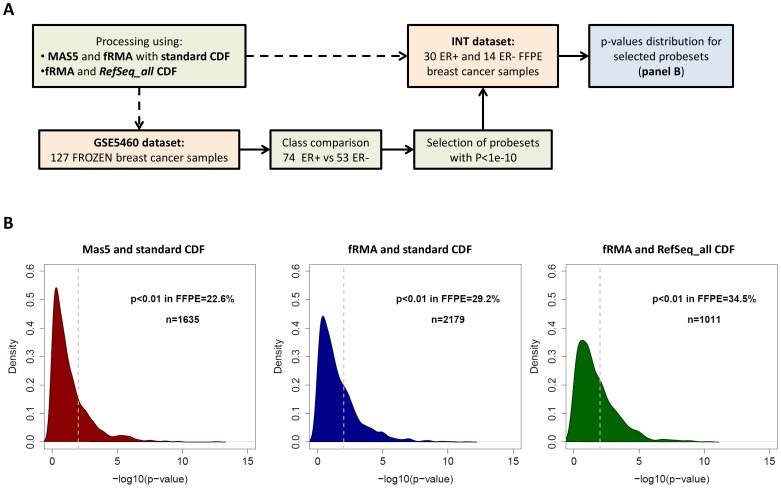
Correlation between frozen- (FFN) and FFPE-derived fold changes as a function of the processing procedure. Fold changes between ABC and GCB subgroups were computed in the Williams dataset [15] for three representative processing pipelines, separately for frozen- and FFPE-derived data. Commonly DE probesets are in dark yellow, probesets only DE in frozen data are in blue and those only DE in FFPE data are in dark red.

The impact of the normalization method (MAS5 vs fRMA) and type of CDF (standard vs *RefSeq_all*) was graphically represented in Figure 1. Notably, the data processing has an influence also on frozen data. Indeed, besides the fraction of commonly DE probesets, the percentage of DE probesets in frozen data rose from 0.7% (396/54675) in MAS5 normalized data to 1% (554/54675) in fRMA normalized data with the standard CDF.

Finally, results in Table 2 and Figure 1 lead to the observation that FFPE data, also with the best performing method, still suffers from a false negative problem, in fact they have a significantly lower power. However generally speaking what is identified in FFPE data is mostly confirmed in frozen data.

### Independent confirmation of data processing relevance

We sought to confirm the findings generated in the Williams dataset using two independent breast cancer datasets: the GSE5460 dataset [24], derived from 127 frozen breast cancers and a dataset of 44 FFPE breast cancers profiled in our institution (hereafter called INT dataset, see Materials and Methods for details).

**Figure 2:**
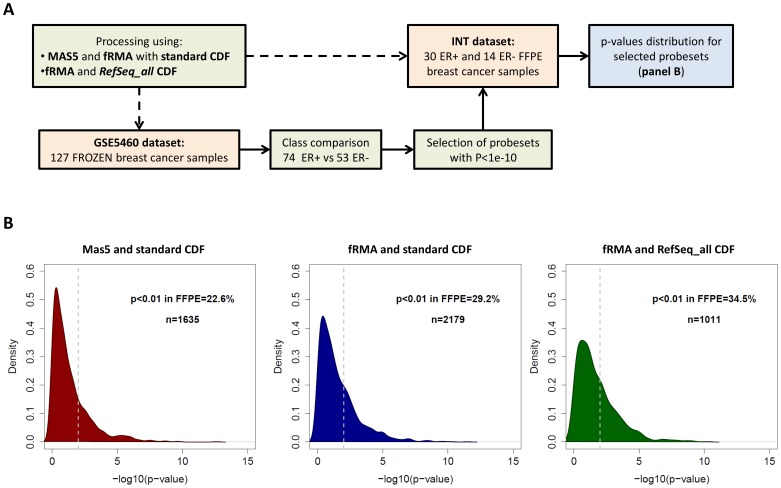
Sensitivity of FFPE data after applying different processing pipelines in two breast cancer datasets. (A) Flow chart of the analysis. (B) Distribution of p-values in the INT FFPE dataset for ER-related probesets identified in the GSE5460 frozen dataset. The analysis was performed on data processed using MAS5 and the standard CDF (left), fRMA and the standard CDF (center) or fRMA and the *RefSeq_all* CDF (right).

Through the analysis described in Figure 2A, we aimed to confirm that data processing affects the capability of identifying truly DE genes in FFPE-derived data. The two datasets were processed using MAS5 or fRMA with the standard CDF or fRMA with the *RefSeq_all* CDF. Class comparison between estrogen receptor (ER) positive and negative tumors was performed in the GSE5460 frozen dataset and probesets with *P*<1e-10 were selected. Genes targeted by such probesets, identified using a very stringent *P*-value threshold and a large dataset of GEPs from frozen tissues can be reasonably considered as true ER related genes. As reported in Figure 2B, the *P*-values computed in the INT FFPE dataset for these “gold standard” probesets significantly shifted towards lower values when using fRMA instead of MAS5 in combination with the standard CDF (fRMA standard *vs* MAS5 standard, *P* = 1.18e-08), with a percentage of probesets having *P*<0.01 rising from 22.6% to 29.2%. At the same time, an improvement seems to happen also in frozen data as the total number of DE probesets increased from 1635 to 2179 out of 54675, corresponding to the 3.0% and 4.0% respectively. The use of the *RefSeq_all* CDF with fRMA further shifted the *P*-value distribution (fRMA *RefSeq_all* vs fRMA standard, *P* = 1.23e-04) and 34.5% of the “gold standard” probesets had *P*<0.01 in FFPE data. Overall these results also suggest that despite using an optimized processing algorithm, many genes will not be detected as DE when using GEPs derived from FFPE material. This observation should be carefully considered when data generated from frozen-material has to be validated in data derived from FFPE.

**Figure 3:**
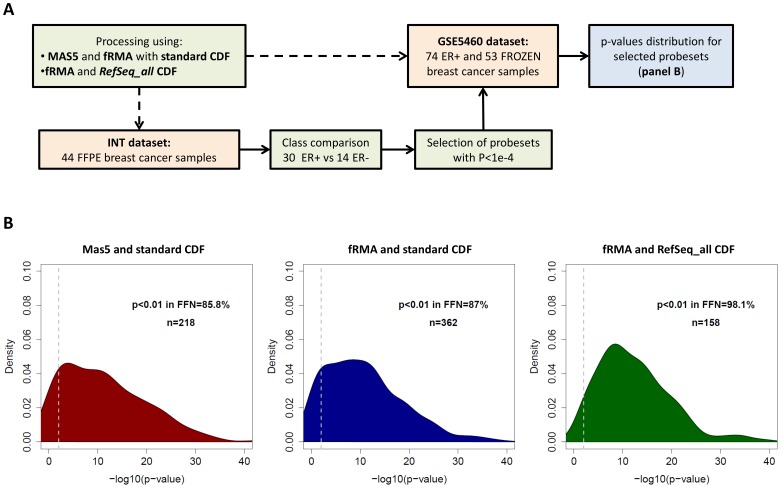
Evaluation of the positive predictive value of FFPE data after applying different processing pipelines in two breast cancer datasets. (A) Flow chart of the analysis. (B) Distribution of p-values in the GSE5460 frozen dataset for probesets DE in the INT FFPE dataset between ER+ and ER− tumors. The analysis was performed on data processed using MAS5 and the standard CDF (left), fRMA and the standard CDF (center) or fRMA and the *RefSeq_all* CDF (right).

Using the same datasets, we also employed FFPE-derived data to identify the genes DE between tumor subtypes and then evaluated the proportion of these genes which can be confirmed as DE in frozen derived GEPs according to different processing methods. To this aim we performed the analysis as described in Figure 3A. Class comparison between ER positive and negative tumors was performed in the INT FFPE dataset and the probesets with *P*<1e-4 were defined as DE. Their *P*-value distribution in the frozen dataset were similar when using MAS5 or fRMA in combination with the standard CDF (Figure 3B), with a percentage of probesets having a *P*<0.01 equal to 85.8% and 87.0% respectively (MAS5 standard vs fRMA standard, *P* = 0.62). However, the number of probesets identified as DE in the FFPE dataset were higher for fRMA compared to MAS5, 362 (0.7%) and 218 (0.4%) respectively. The use of the *RefSeq_all* CDF further increased the proportion of DE probesets (up to 0.9%) and the *P*-value distribution in frozen data shifted towards lower values (fRMA *RefSeq_all* vs fRMA standard, *P* = 0.016; fRMA *RefSeq_all* vs MAS5 standard, *P* = 0.108), with 98.1% of probesets having *P*<0.01 in frozen data.

In the previous analysis no correction for multiple testing was applied; however similar results were obtained by selecting as DE probesets in FFPE data those with a Benjamini-Hochberg adjusted p<0.01 (Figure S4).

**Figure 4:**
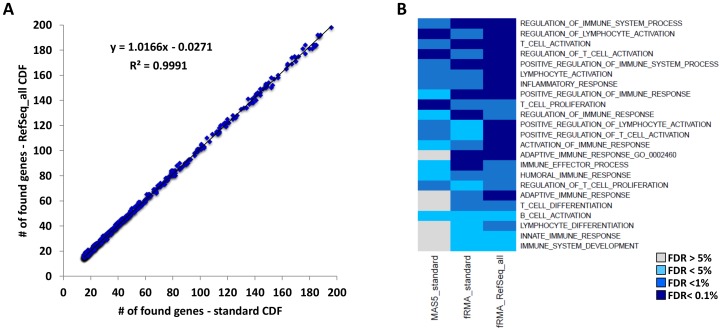
Immune gene set enrichment analysis results from the comparison of samples with and without lymphocitic infiltration in the INT FFPE dataset. (A) Number of genes composing each gene set that was found in the standard CDF compared with the number of genes found in the *RefSeq_all* CDF. (B) Heatmap representing positive enrichment significance for the immune gene sets after processing the data with three different pipelines.

Threshold criteria adopted in the generation of the alternative CDF could have the drawback of losing potentially informative markers, considering that about 2000 less genes are evaluated when using the *RefSeq_all* CDF compared to the standard CDF (Table 1). To verify whether it can have an impact when a biological interpretation is of interest, we performed a gene set enrichment analysis in the INT FFPE dataset between tumors having or not lymphocytic infiltration. Since multiple probesets per gene are not allowed in this analysis, for the standard CDF one probeset per gene was selected according with the method developed by Li et al. [23], somehow selecting the best performing probesets. Of the genes composing each gene set in the C5 MSigDB collection (gene ontology gene sets), the number of those actually found (i.e. assayed) in data processed using either the alternative CDF or the standard CDF was quite identical (Figure 4A), suggesting that genes lost in the alternative CDF are frequently poorly characterized genes determining a minimal impact on pathway analyses. Moreover, a set of immune-related gene sets was selected and tested for enrichment in expression data processed with the same three pipelines described before.

In the comparison of breast cancers with and without lymphocytic infiltration, the selected immune gene sets were similarly enriched in fRMA normalized data, with a trend of higher enrichments using the *RefSeq_all* CDF, while, again, poorer results were obtained in the MAS5 normalized data (Figure 4B).

### Tools availability

Globally the use of an alternative CDF in combination with the fRMA algorithm seems to be advantageous in many situations. The parameters required by fRMA have to be re-estimated if using an alternative CDF and this is time consuming and a computationally demanding task. To make available an immediately applicable bioinformatic tool, the software and data files needed to implement the pipelines using an alternative CDF are available at: http://www.biocut.unito.it/cdf-ffpe/.. *RefSeq_all* and *RefSeq_dist* aCDFs were generated for Affymetrix HG-U133a, Affymetrix HG-U133b and HG-U133 Plus 2.0 chips. Re-estimated parameters for each combination of chip (U133a and Plus 2.0) and CDF are also available. Moreover, sample R code was included. Updated CDFs (and related fRMA parameters) using new RefSeq versions will be periodically released.

## Conclusions and Discussion

Obtaining reliable gene expression data from fragmented RNA derived from FFPE specimens is challenging, with many pre-analytical and analytical steps which can affect the quality of the results [7], [16]. However, the approach is feasible and it has been proved to be able to generate valuable data for translational studies [10], [12]. In this study we demonstrated that also an appropriate data processing can significantly improve the reliability of gene expression data derived from FFPE tissues and generated using the Affymetrix platform.

Our methodology was specifically developed for the Affymetrix chips of the HG-U133 series. While microarrays in general, and the HG-U133 series in particular, can be considered an obsolete way of conducting gene expression profiling experiments, the availability of an impressive corpus of data obtained with this platform on many different tumors makes this choice still appealing by making comparisons with previous results straightforward. In fact these chips are still used in current projects [10]–[13], [25] and improving the methods for their analysis is still relevant.

By assessing the performance of different processing approaches and comparing distinct molecular groups in both lymphoma and breast cancer (ABC vs GCB or ER+ vs ER−), we identified the use of fRMA as normalization method in combination with the *RefSeq_all* CDF as the more effective processing approach. The advantage of using this optimized processing could be even more valuable in the identification of the tiny differences expected in biomarker discovery for more demanding prognostic and predictive clinical questions in the modern era of personalized medicine. Indeed, our algorithm has been successfully applied to GEPs derived from a large series of FFPE tumor samples from patients enrolled in the ECTO randomized clinical Phase III trial [26], where GEPs have been used to predict outcome after adjuvant/neoadjuvant chemotherapy [27].

## Methods

### Datasets

All datasets used in this study contained data from the Affymetrix HG-U133 Plus 2.0 microarray platform and are available on GEO repository [22]. Table S3 summarizes their features.

*GSE19246* [15] – contains 59 matched frozen and FFPE DLBCL patient samples. The dataset contained both prognostic subgroups of DLBCL: germinal center B-cell (i.e. GCB) and activated B-cell (i.e. ABC). Frozen samples were amplified using both the traditional Eberwine oligo-dT method and the Nugen WT-Ovation FFPE System, but only the second were used in our analysis, as this was the amplification method for the matching FFPE samples. After a quality control assessment, three cases (IDs: A6, B3, E6) were excluded from the analysis due to poor quality of FFPE data (low present call and low correlation with the other profiles).

*GSE5460* [24] – contains expression data from 127 frozen breast cancer samples hybridized using the standard Affymetrix protocol. Of them, 74 were estrogen receptor positive (ER+) and 53 ER−.

*GSE38554* (INT dataset) – contains expression data from 44 FFPE primary breast cancer samples taken for routine diagnostic purposes in our Institution between 1997 and 2002. The patients signed an informed consent and this study was approved by the Institutional Review Board and independent ethic committee of Fondazione IRCCS Istituto Nazionale dei Tumori (INT). Thirty samples were from ER+ and 14 from ER− tumors. Presence of lymphocytes infiltration (LI) was quantified by an expert pathologist. Tumors having 5% or less LI (n = 22) were compared with those having 10% or more LI (n = 22). RNA was amplified with the Nugen WT-Ovation FFPE System. A quality control was performed at each level, from sample representativeness to expression profile. Methodological details will be described in an independent manuscript submitted for publication.

### Alternative CDFs and normalization

Alternative CDFs were generated as previously described [28]. Briefly, the sequences of all human mRNAs included in RefSeq database (hg19, GRCh37) were obtained from the UCSC genome browser. For genes with multiple RefSeq transcripts, the longest isoform was considered. These isoforms were then used as input for the *altcdfenvs* Bioconductor package (version 2.16.0, R version 2.14.1) [20], [29], which generates the alternative CDF. New probesets were required to contain at least 5 uniquely mapped probes (*RefSeq_all* CDF), otherwise were not further considered. For the *RefSeq_dist* CDF the five probes closest to the 3′-end and in any case mapping within 300 bp from the 3′-end were used.

The *affy* Bioconductor package (version 1.32.1) [30] was used for data import and MAS5 and RMA normalization, while for fRMA we used the package of the same name (version 1.6.0) [19]. fRMA is based on pre-computed parameters estimated using a large set of CEL files derived from the GEO repository [22]. As parameters need to be re-estimated when using an alternative CDF, we carried out our own estimation using the same set of 200 batches of 5 samples used by the authors, taking advantage of the functions implemented in the *frmaTools* Bioconductor package (version 1.6.0) [31].

Standard probesets were annotated using the *hgu133plus2.db* Bioconductor package (version 2.6.3) and redefined probesets were annotated using the *org.Hs.eg.db* Bioconductor package (version 2.6.4)

### Statistical analysis

Pearson's correlation was used to compute samples, probeset and fold change correlation analysis in the GSE19246 dataset.

Student's t-test was used to assess probesets differential expression. Probesets with p<1e-4 were defined as DE if not otherwise specified.

Differences in p-value distributions were tested for significance using the Wilcoxon-Mann-Whitney test.

Enrichment analysis was performed using GSEA (v. 2.0) [32]. From the C5_BP collection (v. 3.1) containing 825 gene sets derived from the Biological Process Gene Ontology, immune related gene sets were selected searching for the following keyword in their names: immune, lymphocytes, inflammatory, T cell and B cell. A total of 30 gene sets were selected and only those for which a number of genes >15 and <200 was found in the data were tested. Gene sets with FDR<5% were considered significantly enriched.

## Supporting Information

Figure S1 (A) Number of probes in each probeset in the *RefSeq_all* CDF. (B) RNA degradation plot for the frozen (left) and FFPE (right) data in the Williams dataset [15]. For each chip, probe intensities are averaged by location in probeset, with the average taken over probesets.
(TIF)

Figure S2 Frozen-FFPE correlation as a function of the distance of the probes from 3′-end. (A) Frozen-FFPE correlation for the 56 matched samples of the GSE19246 dataset increases when only probes nearer to the 3′-end are selected. (B) Frozen-FFPE pair plot of probe-level log2 intensities for a representative sample. Probes with a 3′-distance <100 bp are highlighted in red.
(TIF)

Figure S3 Average distribution of signals in the FFPE GSE19246 dataset (n = 56) for the 8263 common genes using *RefSeq_all* and *RefSeq_dist* CDFs and fRMA normalized data.
(TIF)

Figure S4 Evaluation of the positive predictive value of FFPE data after applying different processing pipelines in two breast cancer datasets. (A) Flow chart of the analysis. (B) Distribution of p-values in the GSE5460 frozen dataset for probesets DE (FDR<1%) in the INT FFPE dataset between ER+ and ER− tumors. The analysis was performed on data processed using MAS5 and the standard CDF (left), fRMA and the standard CDF (center) or fRMA and the *RefSeq_all* CDF (right).
(TIF)

Table S1 Wilcoxon matched pairs test of frozen-FFPE sample correlations using different processing pipelines.
(XLSX)

Table S2 Mann-Whitney test of frozen-FFPE probesets correlations using different processing pipelines.
(XLSX)

Table S3 Main features of the datasets analyzed.
(XLSX)
