# Correction: Accurate Data Processing Improves the Reliability of Affymetrix Gene Expression Profiles from FFPE Samples

**DOI:** 10.1371/journal.pone.0095814

**Published:** 2014-04-15

**Authors:** The PLOS ONE Staff

Figure 1 is incorrect and has been updated here.

Please see the corrected Figure 1.

**Figure 1:**
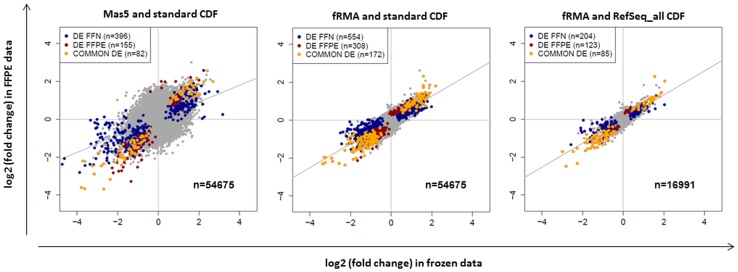
Correlation between frozen- (FFN) and FFPE-derived fold changes as a function of the processing procedure. Fold changes between ABC and GCB subgroups were computed in the Williams dataset [15] for three representative processing pipelines, separately for frozen- and FFPE-derived data. Commonly DE probesets are in dark yellow, probesets only DE in frozen data are in blue and those only DE in FFPE data are in dark red.
