# Supplementary figures and images for: Accurate Data Processing Improves the Reliability of Affymetrix Gene Expression Profiles from FFPE Samples
Source: PLoS One. 2014 Jan 29;9(1):e86511. doi: 10.1371/journal.pone.0086511 (PMC3906036; doi:10.1371/journal.pone.0086511)

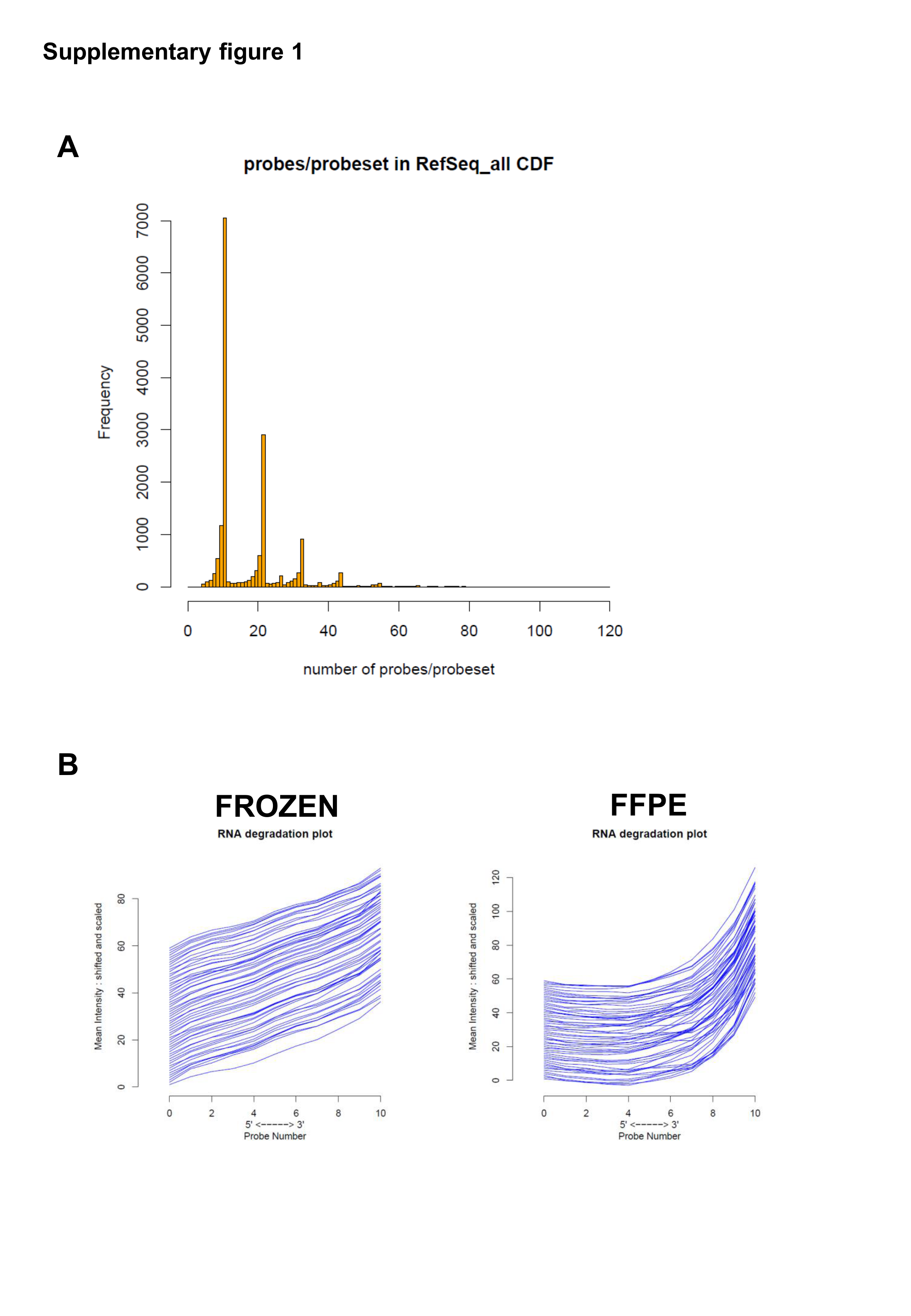

Supplement: Figure S1 — (A) Number of probes in each probeset in the RefSeq_all CDF. (B) RNA degradation plot for the frozen (left) and FFPE (right) data in the Williams dataset [15]. For each chip, probe intensities are averaged by location in probeset, with the average taken over probesets. (TIF) [file pone.0086511.s001.tif]

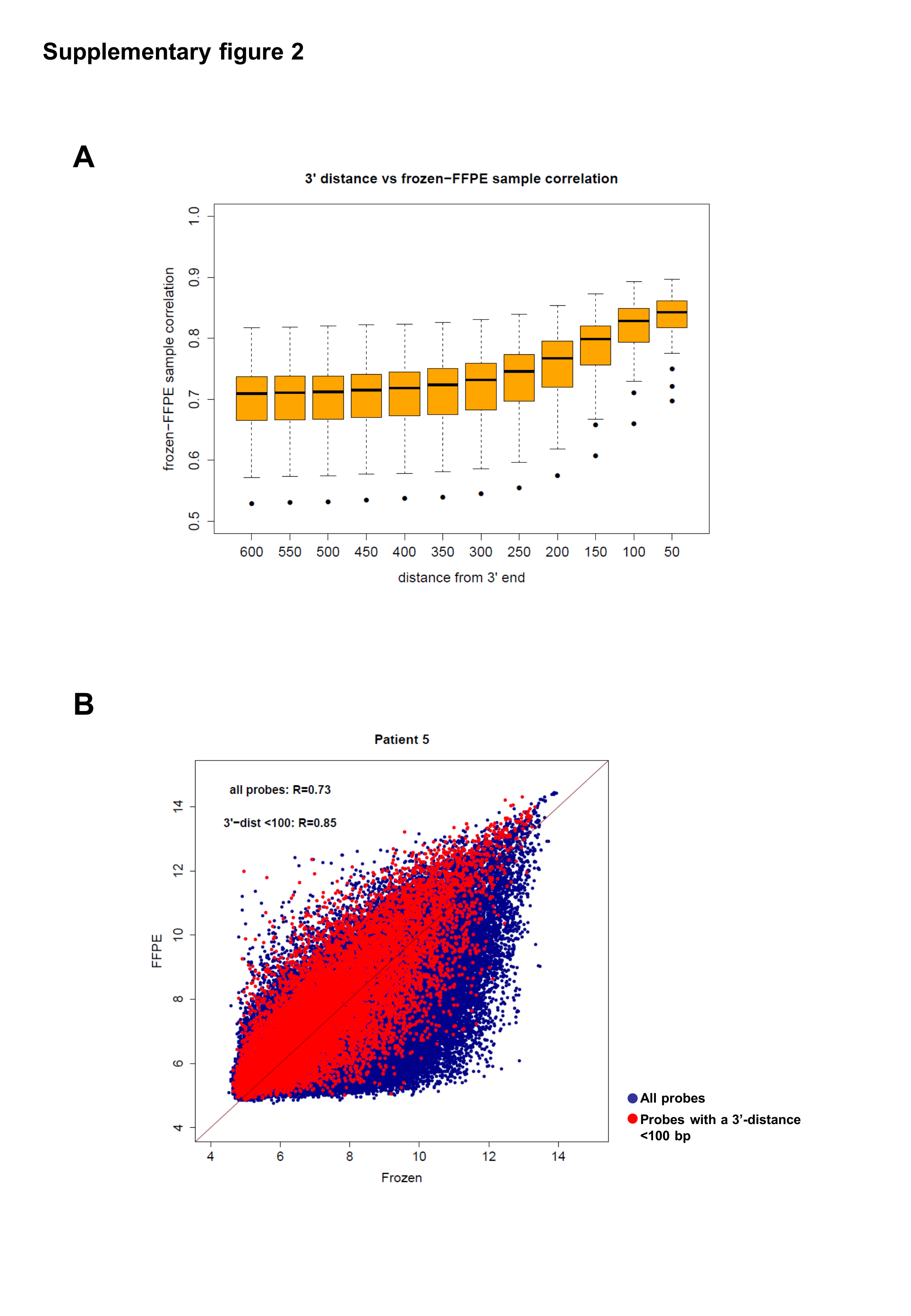

Supplement: Figure S2 — Frozen-FFPE correlation as a function of the distance of the probes from 3′-end. (A) Frozen-FFPE correlation for the 56 matched samples of the GSE19246 dataset increases when only probes nearer to the 3′-end are selected. (B) Frozen-FFPE pair plot of probe-level log2 intensities for a representative sample. Probes with a 3′-distance <100 bp are highlighted in red. (TIF) [file pone.0086511.s002.tif]

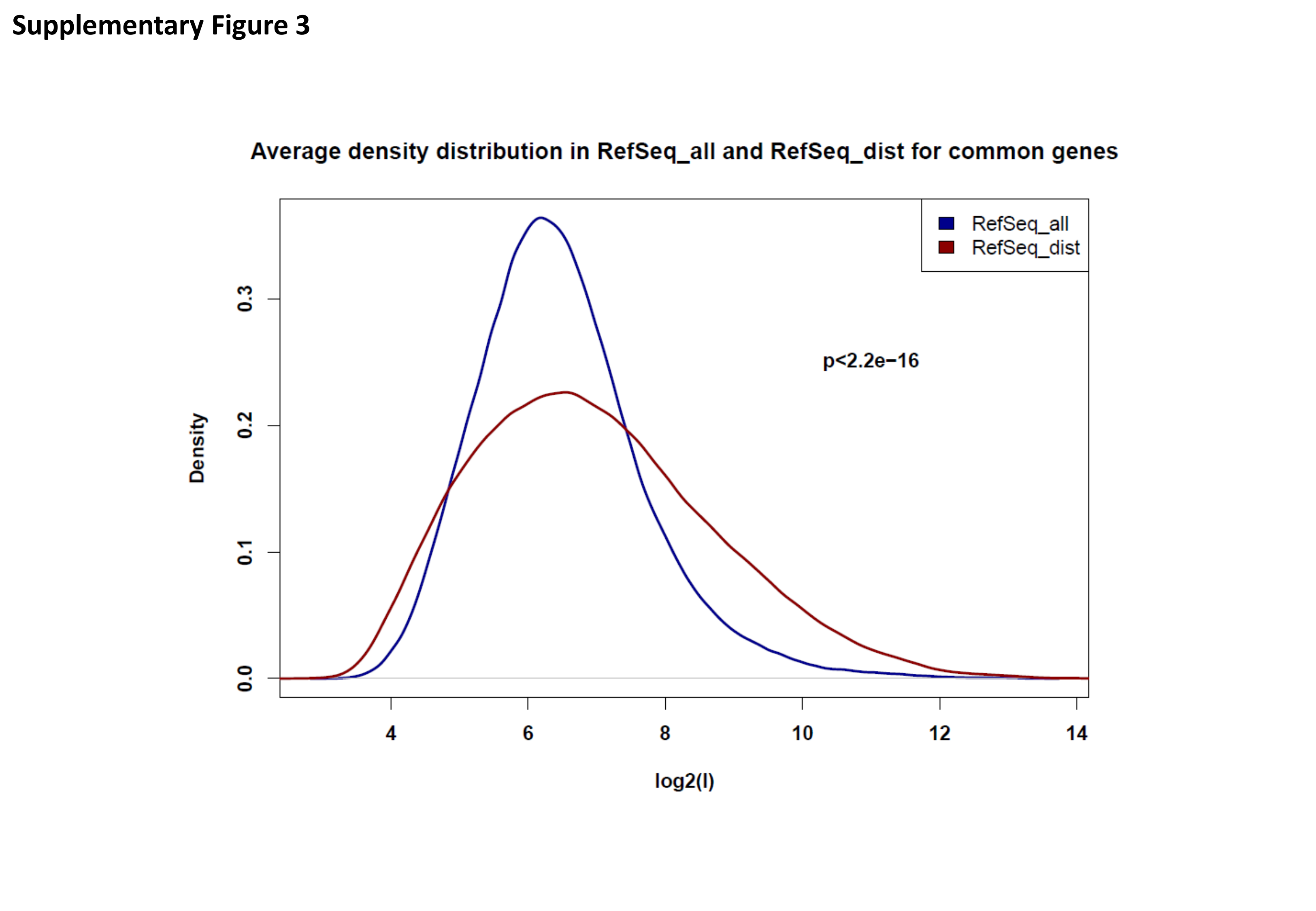

Supplement: Figure S3 — Average distribution of signals in the FFPE GSE19246 dataset (n = 56) for the 8263 common genes using RefSeq_all and RefSeq_dist CDFs and fRMA normalized data. (TIF) [file pone.0086511.s003.tif]

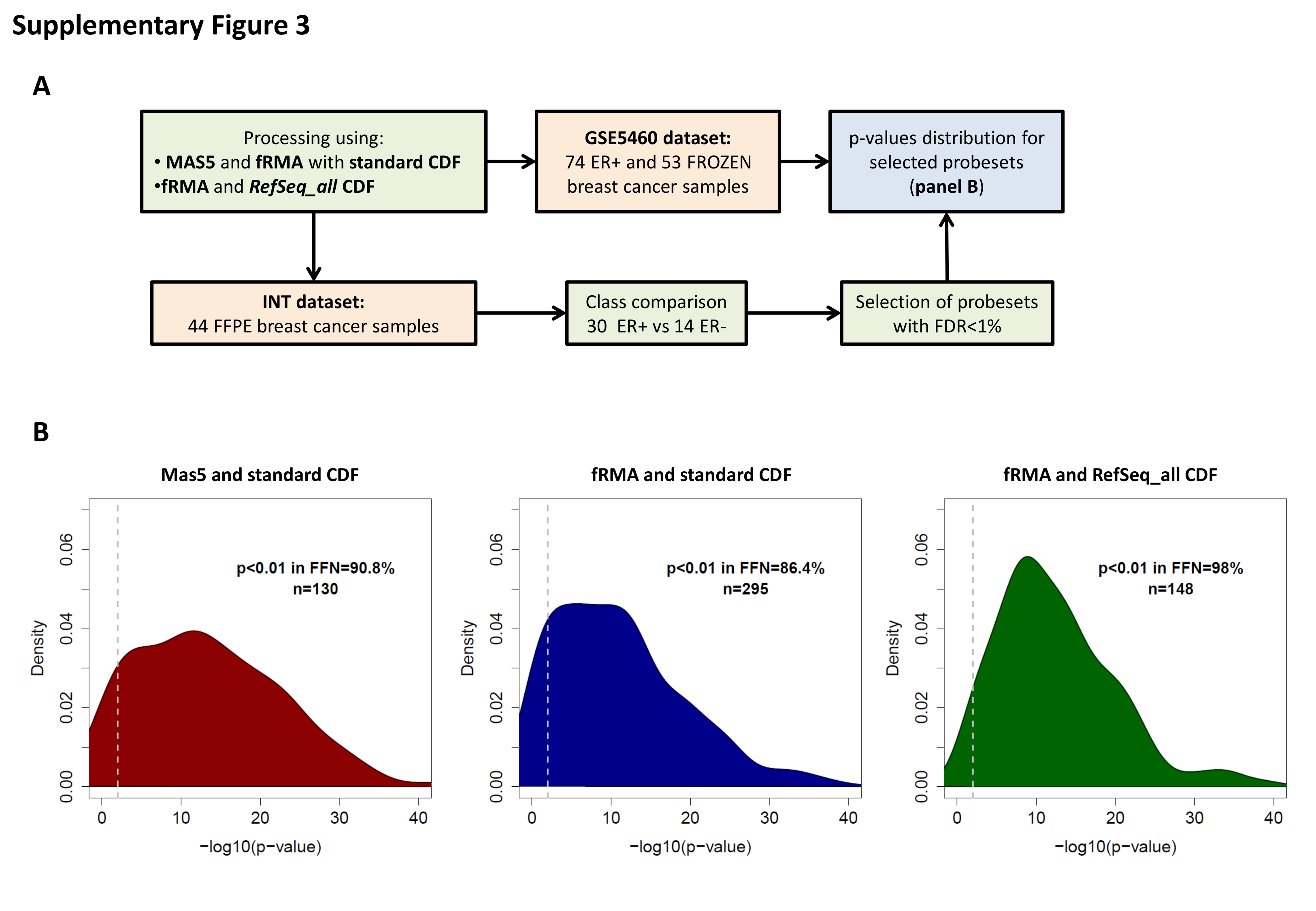

Supplement: Figure S4 — Evaluation of the positive predictive value of FFPE data after applying different processing pipelines in two breast cancer datasets. (A) Flow chart of the analysis. (B) Distribution of p-values in the GSE5460 frozen dataset for probesets DE (FDR<1%) in the INT FFPE dataset between ER+ and ER− tumors. The analysis was performed on data processed using MAS5 and the standard CDF (left), fRMA and the standard CDF (center) or fRMA and the RefSeq_all CDF (right). (TIF) [file pone.0086511.s004.tif]
